# In Vitro Antimicrobial Activity of Chlorhexidine–Curcumin Nanoformulations for Teat Dipping Against Mastitis Pathogens

**DOI:** 10.3390/vetsci13101054

**Published:** 2026-10-08

**Authors:** Areerat Chuasakhonwilai, Wasana Chaisri, Montira Intanon, Duanghathai Saipinta, Patsorn Keeklangdon, Pimnipa Jieraviriyapun, Sho Nakamura, Satoshi Ohkura, Witaya Suriyasathaporn

**Affiliations:** 1 Faculty of Veterinary Medicine, Chiang Mai University, Chiang Mai 50100, Thailandwasana.ch@cmu.ac.th (W.C.); duanghathai.s@cmu.ac.th (D.S.);; 2 Office of Research Administration, Chiang Mai University, Chiang Mai 50200, Thailand; 3 Research Center of Producing and Development of Products and Innovations for Animal Health and Production, Chiang Mai University, Chiang Mai 50100, Thailand; 4 Laboratory of Animal Production Science, Graduate School of Bioagricultural Sciences, Nagoya University, Nagoya 464-8601, Japan; 5 Asian Satellite Campuses Institute, Nagoya University, Overseas Campus, Nagoya 464-8601, Japan

**Keywords:** antimicrobials, mastitis pathogens, curcumin chlorhexidine, nanoparticle, teat dip

## Abstract

Bovine mastitis is a major problem affecting dairy production, and teat disinfection is important for reducing the risk of bacterial infection. Curcumin has antimicrobial properties, but its poor water solubility limits its use in aqueous formulations. This study aimed to develop chlorhexidine–curcumin nanoformulations (CCNs) and perform preliminary in vitro screening of these nanoformulations as candidate teat-dipping formulations. Four CCNs containing increasing concentrations of curcumin (0%, 0.1%, 0.5%, and 0.9% *w*/*v*; CCN0, CCN0.1, CCN0.5, and CCN0.9, respectively) were prepared. CCN0, CCN0.1, and CCN0.5 were formulated as oil-in-water nanoemulsions, whereas CCN0.9 was prepared as a curcumin-β-cyclodextrin inclusion complex. The physicochemical properties were characterized, and the antibacterial activity was evaluated in vitro by agar well diffusion against six mastitis-associated pathogens. CCN0.9 showed the largest inhibition zones overall (20.07 ± 1.05–28.85 ± 1.05 mm; *p* < 0.05), while CCN0.1 and CCN0.5 showed activity comparable to or greater than that of commercial teat dips against several pathogens. CCN0.5 showed the most favorable nanoemulsion characteristics, with a particle size of 94.77 nm and a zeta potential of −46.57 mV. Overall, CCNs demonstrated antibacterial activity against mastitis pathogens, supporting their potential as candidate teat-dip formulations. Further teat irritation and in vivo efficacy studies are needed before field application.

## Simple Summary

Bovine mastitis is among the most significant diseases affecting the dairy industry, and teat dipping after milking is important for controlling new intramammary infections. Most commercial teat dips contain disinfectants, but excessive use raises concerns about residues in milk and multidrug-resistant pathogens. Curcumin has anti-inflammatory properties but poor water solubility. This study evaluated the in vitro inhibitory activity of four chlorhexidine–curcumin nanoformulations containing different curcumin concentrations against mastitis-associated bacteria and compared their activity with that of commercial teat-dipping products. The results revealed that the antibacterial activity of the nanoformulation was comparable to or greater than that of commercial products against selected bacteria in an agar well diffusion assay. However, these findings do not establish teat-skin safety or practical teat-disinfection efficacy. The safety and effectiveness of these formulations needed to be further evaluated.

## 1. Introduction

Bovine mastitis is an inflammation of the mammary gland and is among the most significant causes of economic loss in the dairy industry [1] because of reduced milk production and quality and increased culling [2]. Mastitis is caused by intramammary bacterial infections, with *Escherichia coli*, *Staphylococcus aureus*, *Streptococcus uberis*, and *Streptococcus agalactiae* being the major pathogens associated with clinical mastitis. Nonaureus staphylococci (NASs), such as *Staphylococcus hominis* and *Staphylococcus chromogenes*, are common etiological agents of subclinical mastitis and are considered minor pathogens [3].

The National Mastitis Council recommends a comprehensive mastitis control program that includes prevention and treatment [4]. Teat dipping has been widely recognized as an effective strategy for preventing intramammary infections by killing mastitis pathogens before they cause these infections [5]. Commercial products use disinfectants with antimicrobial properties such as iodine, chlorine compounds, and hydrogen peroxide [6,7]. The increasing use of antiseptic teat dipping on dairy farms has raised concerns about potential adverse effects, particularly residues in dairy products and their associated risks to human health [8,9]. Prolonged exposure to these antiseptic compounds exerts selective pressure on microbial populations, thereby facilitating the emergence of multidrug-resistant pathogens, which represents a significant challenge to both animal and public health [7]. However, among disinfectants, chlorhexidine helps address this public health problem by maintaining consistent efficacy against both *E*. *coli* and *S*. *aureus* and not showing an increasing trend in minimum inhibitory concentration (MIC) over time [7].

On farms with poor mastitis control programs, improper milking machines and milking procedures can damage teat ends and cause small wounds, increasing the risk of mastitis [10]. Multipurpose teat dipping should be appropriate for both preventing new IMIs and promoting teat-end wound healing. However, most commercial teat-dipping antiseptics help prevent new infections and have moisturizing properties for teats but do not accelerate wound healing. Curcumin has wound-healing and anti-inflammatory properties, helping reduce reactive oxygen species levels and promoting collagen deposition and granulation tissue formation [11]. Although curcumin or turmeric extract is a natural antibacterial agent [12,13], very high concentrations are needed to kill mastitis pathogens [6]. The combination of curcumin with various antiseptics reduced antimicrobial activity, however, the efficacy remained satisfactory when combined with chlorhexidine [6,14]. Despite its potential, the efficacy of curcumin is constrained by its physicochemical properties, particularly its poor water solubility and low bioavailability [15,16]. To address these challenges, previous studies have explored nanotechnology-based delivery strategies, such as nanoemulsions and cyclodextrin inclusion complexes, to improve the physicochemical properties and biological activities of curcumin [17,18]. However, these approaches have focused primarily on the use of curcumin alone to increase its solubility, stability, and bioactivity; however, the application of curcumin in combination with conventional antiseptic agents and specifically for teat disinfection remains insufficiently investigated.

This study aimed to develop curcumin-based nanoformulations, including a nanoemulsion and a cyclodextrin inclusion complex, in combination with the antiseptic agent chlorhexidine. We hypothesized that chlorhexidine–curcumin nanoformulations would retain antibacterial activity against mastitis-associated bacteria while improving the aqueous dispersion and physicochemical properties of curcumin in a teat-dip formulation. The novelty of this study lies not only in formulating curcumin at the nanoscale but also in developing and evaluating these formulations for specific veterinary applications, namely, teat disinfection in dairy cattle. Particular attention was given to whether the nanoformulation could maintain or enhance the antimicrobial activity of the combined agents while providing physicochemical characteristics suitable for practical application.

## 2. Materials and Methods

This study was performed as two experiments. Nanoformulation is used as the general term for the two delivery systems, namely, the nanoemulsion and cyclodextrin inclusion complex. Experiment 1 (Exp 1) involved the development of chlorhexidine–curcumin nanoformulations for teat dipping and the evaluation of their physicochemical characteristics, and Experiment 2 (Exp 2) involved the evaluation of the antimicrobial activity against mastitis pathogens of all the formulations compared with that of 2 commercial teat-dipping antiseptics. In Exp 1, four chlorhexidine–curcumin nanoformulations (CCNs), including 3 nanoemulsions and 1 cyclodextrin inclusion complex, were prepared according to studies by Mishra and colleagues [19] and Rachmawati and colleagues [20], respectively, with modifications for teat dipping (Table 1). The 3 nanoemulsion formulations were prepared at curcumin concentrations of 0, 0.1, and 0.5% as CCN0, CCN0.1, and CCN0.5, respectively, while the cyclodextrin inclusion complex was prepared at a curcumin concentration of 0.9%, as CCN0.9. For teat dipping purposes, all formulations were supplemented with 5% glycerin. The active ingredients in each formulation are listed in Table 1. All the formulations were then reproduced in triplicate, and their physicochemical characteristics were evaluated separately for each production.

Exp 2 followed the timeline for triplicate production of the chlorhexidine–curcumin nanoformulation. Two commercial teat-dipping antiseptics, one from a worldwide company (TeatDip1, Evans Vanodine International, Preston, UK), an iodophor-based teat dip (0.5% *w*/*v* available iodine), and one from a local company in Thailand (TeatDip2, Farm tech Co., Ltd., Bangkok, Thailand), with an iodine-based teat dip (3% *w*/*v* available iodine), were purchased and used as control solutions. Both products were ready-to-use formulations and were used directly according to the manufacturer’s instructions. Before use, the products were stored at room temperature in accordance with the manufacturer’s instructions, and five strains of each bacterial species were evaluated. For *S. aureus*, *E. coli*, *S. uberis*, and *S. agalactiae*, one reference strain and four field strains were included, whereas five field strains each were included for *S. hominis* and *S. chromogenes.* The reference strains were *S. aureus ATCC 25923*, *E. coli ATCC 25922*, *S. uberis ATCC 27958,* and *S. agalactiae ATCC 27956.* The field strains were previously obtained from subclinical mastitis-affected milk samples from dairy cows on smallholder dairy farms in Chiang Mai, Thailand, and were identified and confirmed with a MALDI-TOF mass spectrometer system (MALDI ID Plus, Shimadzu Corporation, Kyoto, Japan). Briefly, a single colony of each isolate was smeared onto a target plate, overlaid with 1 µL of α-cyano-4-hydroxycinnamic acid (CHCA), and air dried before spectral acquisition and database matching. Confirmed isolates were subsequently stored in the laboratory for milk quality and mastitis control at the Faculty of Veterinary Medicine, Chiang Mai University, Chiang Mai, Thailand, until further use.

### 2.1. Preparation of Chlorhexidine–Curcumin Nanoformulations for Teat Dipping

Briefly, the CCN0, CCN0.1, and CCN0.5 nanoemulsions were prepared as three separate phases at 50 °C. The oil phase consisted of curcumin solutions at different concentrations (0, 0.1, and 0.5% *w*/*v*), 0.38% *w*/*v* β-cyclodextrin, and 10% liquid paraffin, while the surfactant phase consisted of 2% Span 80 and 1% dimethyl sulfoxide (DMSO), with continuous stirring until a homogeneous mixture was obtained. The aqueous phase was prepared by dissolving 2% Tween 80 in distilled water. The oil and surfactant phases were mixed until homogeneous. The aqueous phase was then gradually added to the oil-surfactant mixture under continuous stirring for 30 min, resulting in the formation of a coarse emulsion. Afterward, 0.1% chlorhexidine solution was added and mixed well with 5% glycerol. The coarse emulsion was subsequently subjected to probe ultrasonication using a VPX 750 Vibra-Cell ultrasonic processor (Sonics & Materials Inc., Newtown, CT, USA) at 45 °C for 20 min to reduce the droplet size.

For the cyclodextrin inclusion complex, CCN0.9 was formulated in three phases. First, 0.47% *w*/*v* β-cyclodextrin was dissolved in 86.5% distilled water; then, 0.94% *w*/*v* curcumin was dissolved in 4.70% acetone. The two solutions were combined and stirred using a magnetic stirrer at 200 rpm in the dark until a homogeneous mixture was obtained; then, the mixture was sonicated for 5 min at 30 °C with an amplitude of 37% to reduce the particle size and stirred continuously for 5 h to allow complete complexation and solvent evaporation. Finally, 5% glycerol and 0.1% chlorhexidine solution were incorporated as humectant and antiseptic additives, respectively.

### 2.2. Physicochemical Characterization

The particle size, polydispersity index (PDI), zeta potential, viscosity, pH, and electrical conductivity of the prepared formulations were characterized. The particle size, polydispersity index (PDI), zeta potential, conductivity, and viscosity of the four curcumin nanoformulations (CCN0, CCN0.1, CCN0.5, and CCN0.9) were determined by dynamic light scattering (DLS) using a Zetasizer Nano series (Malvern Instruments, Malvern, UK). The pH of the nanoparticles was measured using a digital pH meter (Lab 875; SI Analytics GmbH, Mainz, Germany). The encapsulation efficiency (%EE) of all the formulations was determined following complex formation. A standard stock solution of curcumin 25 μg/mL was prepared. A calibration curves (y = 0.3333x − 0.0000, R^2^ = 1) were obtained over a concentration range of 0.98–15.63 μg/mL. Non-entrapped curcumin was separated from each formulation by centrifugation at 1500 rpm for 10 min. The absorbance of the supernatant was measured at 450 nm using microplate reader (SpectraMax^®^ iD3; Molecular Devices, LLC, San Jose, CA, USA). The percentage of encapsulation efficiency was calculated using the following equation:

%EE = [(Initial added − Non-entrapped) − Initial added] ∗ 100

### 2.3. Antimicrobial Activity Evaluation

The antibacterial properties were examined using the agar well diffusion method as described by Balouiri et al. [21], with modifications. All bacteria from the glycerol stock were recovered in trypticase soy broth (TSB; Himedia, Thane, India) and incubated at 37 °C for 24 h. Each isolate was subsequently subcultured onto selective agar including Edwards medium (ED, Oxoid, Basingstoke, UK) for *Streptococcus* spp., mannitol salt agar (MSA, Himedia, Thane, India) for *Staphylococcus* spp., and MacConkey (Mc, Himedia, Thane, India) agar for *E. coli*. The agar was incubated at 37 °C for 18–24 h. Fresh bacterial cultures were adjusted in 0.85% normal saline solution to a 0.5 McFarland density (10^8^ CFU/mL) and swabbed onto Mueller–Hinton agar (MHA, Himedia, Thane, India). Aseptic wells 6 mm in diameter were cut using a sterile cork borer, and 100 µL aliquots of the test samples were added to each well. The plates were incubated at 37 °C for 18–24 h. Antibacterial activity was determined by measuring the diameter of the clear inhibition zone surrounding each well using digital Vernier calipers. For each bacterial species, five independent isolates were tested, and each isolate was tested in duplicate. The inhibition zone diameters were recorded in millimeters (mm).

### 2.4. Statistical Analysis

All the data are presented as the mean and standard error of the mean (SEM). Comparisons of antimicrobial activity among solutions were performed using a linear mixed effect model (Proc mixed, SAS^®^ OnDemand for Academics version). The inhibition zone diameter was defined as the dependent variable, and the interaction between the tested bacteria and the teat-dipping solutions was defined as the independent variable. The triplicate tests were treated as repeated factors, and the subjects were the bacteria tested for each teat-dipping solution. The correlation structure was type I autoregressive. The least squares means of each level were calculated for comparison. The significance level was defined as *p* < 0.05.

## 3. Results

### 3.1. Physicochemical Characteristics

**Figure 1:**
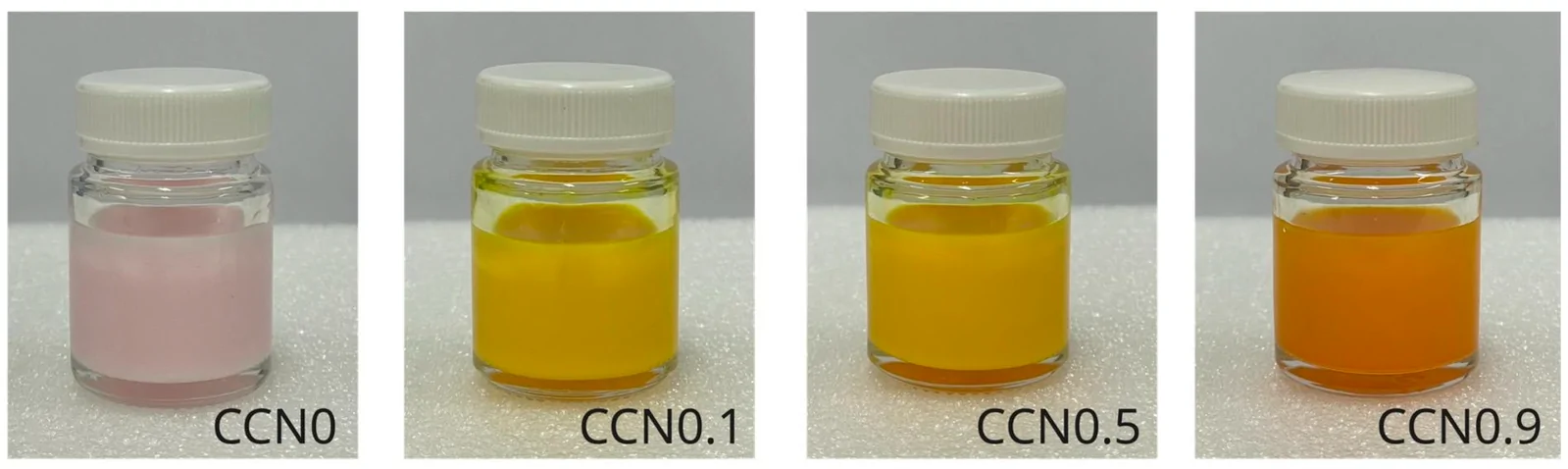
Visual appearance of chlorhexidine-curcumin nanoformulations for teat dipping, identified by curcumin concentrations of 0, 0.1, 0.5, and 0.9% as CCN0, CCN0.1, CCN0.5, and CCN0.9, respectively.

The four CCNs exhibited distinct visual characteristics corresponding to their curcumin content (Figure 1). All the formulations were visually homogeneous, with no apparent sedimentation or phase separation, indicating successful emulsification. CCN0, which was free of curcumin, appeared milky white (Figure 1), whereas the curcumin-containing formulations displayed a gradient of yellow to orange hues that intensified with increasing curcumin concentration.

The particle size, polydispersity index (PDI), zeta potential, pH, and entrapment efficiency varied markedly among the formulations, whereas the viscosity remained comparable across all four formulations (Table 2). CCN0 had the largest droplet size of 2495.7 nm, the highest PDI of 1.64, and a near-neutral zeta potential of −2.2 mV, indicating poor emulsion stability and possible droplet coalescence in the absence of curcumin. In contrast, CCN0.1, CCN0.5, and CCN0.9 exhibited average small-droplet sizes of 101.3, 94.77, and 215.6 nm, respectively, indicating well-formed, physically stable nanoparticles, particularly for the first two solutions. CCN0.1 and CCN0.5 exhibited high absolute zeta potential values of −38.43 and −46.57 mV, respectively, exceeding the ±30 mV threshold required for long-term physical stability against aggregation. Among the tested formulations, CCN0.9 exhibited a larger particle size (215.6 nm) and a broader particle size distribution (PDI = 2.10) than CCN0.5 did (size = 94.77 ± 0.25 nm and PDI = 0.17 ± 0.03, respectively). These results indicate greater particle size heterogeneity in CCN0.9. All the CCN solutions had high encapsulation efficiency (%EE) values of 97.73, 93.9, and 88.57 for CCN0.5, CCN0.9, and CCN0.1, respectively. In terms of physicochemical characteristics, CCN0.5 is the optimal formulation because of its high encapsulation efficiency (97.73%), sub100 nm particle size, excellent polydispersity (PDI = 0.17), and high colloidal stability (Zeta = −46.57).

### 3.2. Antibacterial Activity Against Mastitis-Causing Pathogens

**Figure 2:**
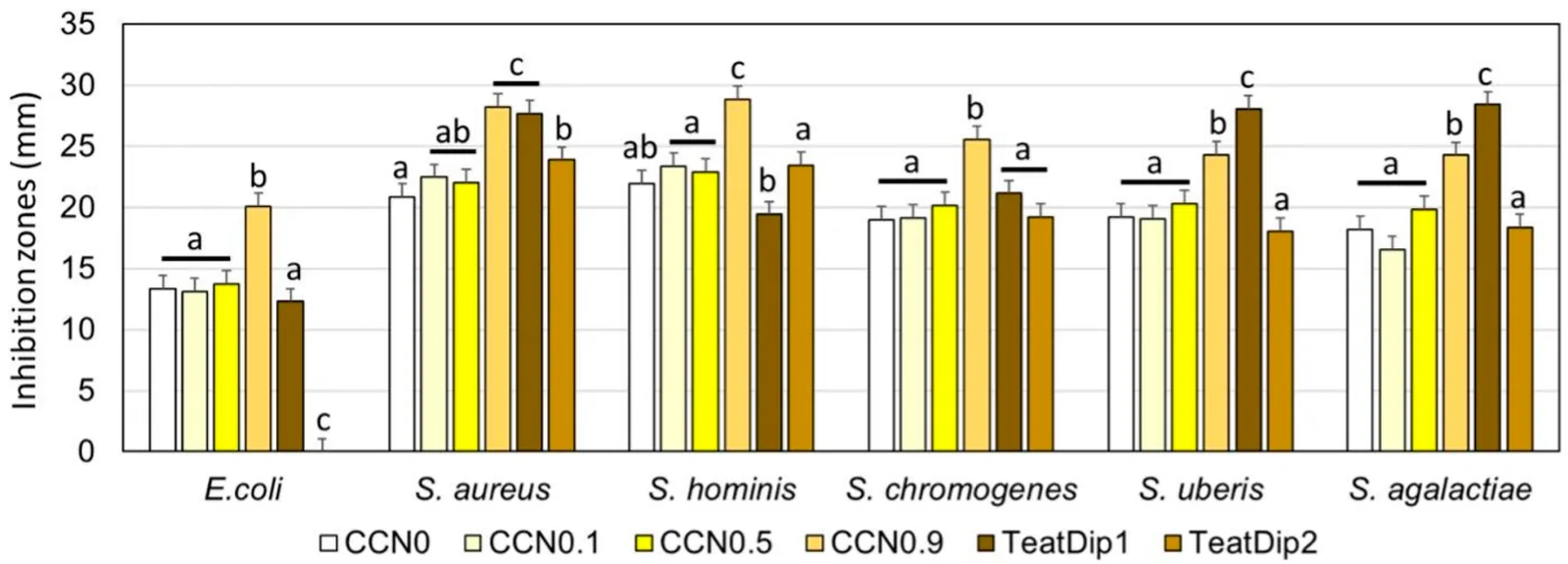
The antibacterial activity of chlorhexidine–curcumin formulations containing 0, 0.1, 0.5, and 0.9% curcumin (CCN0, CCN0.1, CCN0.5, and CCN0.9, respectively) and commercial teat-dipping products (TeatDip1 and TeatDip2) against mastitis pathogens was evaluated by agar well diffusion assays. The data are presented as least-squares means ± standard errors (SEs). Statistical analyses were performed using SAS^®^. Different superscripted letters (a, b, c) indicate significant differences (*p* < 0.05).

The antimicrobial activity results are presented in Figure 2. With the exception of the zero-inhibition zone of TeatDip2 against *E*. *coli* and the larger inhibition zones of TeatDip2 against *S*. *aureus*, CCN0, CCN0.1, CCN0.5, and TeatDip2 had similarly low-level inhibitory zones (Tukey’s test, *p* > 0.05). The 0.9% curcumin formulation produced the largest inhibition zones, particularly against *E*. *coli* (20.07 ± 1.05 mm), *S*. *aureus* (28.24 ± 1.05 mm), *S*. *hominis* (28.85 ± 1.05 mm), and *S*. *chromogenes* (25.53 ± 1.05 mm), while TeatDip1 exhibited the strongest antimicrobial activity against both *S*. *uberis* and *S*. *agalactiae*. Nevertheless, CCN0.9 was the second most effective antimicrobial agent against streptococci. With respect to *E*. *coli*, *S*. *hominis*, and *S*. *chromogenes*, compared with the other groups, the inhibition zones of CCN0.9 were significantly larger, whereas the inhibition zones of both CCN0.9 and TeatDip1 were significantly larger than those of the other groups (*p* < 0.05) for *S*. *aureus*. With respect to streptococci, compared with CCN0.9, TeatDip1 resulted in significantly larger inhibition zones, which in turn resulted in significantly larger inhibition zones in the TeatDip1 group than in the other groups (*p* < 0.05). The results showed that the CCN0.9 formulation exhibited antibacterial activity against the tested mastitis pathogens; however, the contribution of curcumin itself to the observed activity could not be determined because of potential confounding effects related to the formulation and residual solvent.

## 4. Discussion

To formulate a teat-dipping solution, glycerin, an emollient, and chlorhexidine (Table 1) were incorporated into the original curcumin nanoformulations reported by Mishra et al. [19] and Rachmawati et al. [20]. The variations in the characteristics of the nanoformulations in this study might be attributable to several factors involved in formulation conditions, such as the types of substances used, solvent selection, concentration or volume, time, and temperature [22,23]. Our findings show that the droplet sizes of CCN0.1, CCN0.5, and CCN0.9 (Table 2) were within the acceptable nanoparticle range of 20–500 nm [24], whereas CCN0 had a much larger droplet size of 2495.7 nm. Curcumin significantly enhanced interfacial stabilization during emulsification by effectively reducing interfacial tension and working in conjunction with surfactants [25]. In addition, BCD, a cyclic oligosaccharide with a hydrophilic outer surface and a hydrophobic cavity, can interact with hydrophobic compounds such as curcumin [26,27]. Its complexation enhances the aqueous solubility and stability of curcumin, thereby protecting it from degradation [27,28]. However, the large droplet size of CCN0.9 might be related to its higher curcumin concentration than in the other formulations. The more hydrophobic curcumin increases the risk of aggregation and particle-size growth [27,29].

In terms of physicochemical characteristics other than droplet size (Table 2), CCN0.5 is the best chlorhexidine–curcumin nanoformulation, with the highest encapsulation efficiency (97.73%), excellent polydispersity (PDI = 0.17), and the highest colloidal stability (zeta = −46.57). This formulation has similar to or even better nanoemulsion characteristics than the curcumin-annatto oil (size = 263 nm, PDI = 0.15, and zeta = −28.76 mV)) reported by Silva [30] and a curcumin nanoemulsion intended for pulmonary administration (size = 130.6 nm, PDI = 0.151, and zeta = −1.7 mV) [31]. PDI values less than 0.3 are associated with a uniform and consistent droplet population [25]. Absolute zeta values lower than −30 mV or higher than 30 mV indicate sufficient electrostatic repulsion to ensure colloid stability [32]. Although the droplet sizes of CCN0.1 and CCN0.9 were in the nanoparticle size range, PDIs exceeding 0.3 and zeta potentials ranging from 24.4 to 38.4 (Table 2) raise questions, particularly about the homogeneity of their droplet populations and stability. The relatively high PDI of CCN0.9 further indicates a broad and heterogeneous particle-size distribution. High loading of poorly water-soluble compounds can affect droplet formation and the resulting size distribution of a nanoemulsion [33]. Therefore, the physicochemical characteristics of CCN0.9 may reflect the difficulty of maintaining a homogeneous dispersed system at the higher curcumin concentration used in this formulation. However, although these characteristics may affect physical stability, stability was not evaluated in the present study. Further studies investigating different curcumin concentrations and formulation parameters are needed to optimize the formulation and evaluate its long-term stability.

With respect to the encapsulation properties of the nanoemulsion formulations, CCN0.1, CCN0.5, and CCN0.9 had EE percentages of 88.6, 97.7, and 93.9, respectively, indicating acceptable encapsulation efficiencies, as values between 80% and 99% are considered highly successful for the entrapment of curcumin inside the protective matrix or nanocarrier [34]. The acidic pH range of the chlorhexidine-curcumin nanoformulations (CCN0–CCN0.9), which ranged from 4.19 ± 0.282 to 4.68 ± 0.146, is primarily attributed to the presence of chlorhexidine active salts such as chlorhexidine digluconate or acetate. These salts naturally form acidic solutions (pH 4.0–5.5) when dissolved in aqueous or polar media because of the dissociation of weak base–acid salts [35]. Generally, the pH of teat skin decreases from 7.2 two hours before milking to 6.8 two hours after milking, which is greater than that of human skin (4.5–5.5) [36]. However, when an iodine-containing teat dip, approximately pH 2.5–4.5, was used after milking, the pH increased to 7.1 and then decreased to 5.5 [37], similar to the effect of emollient cream in the successful treatment of patients with mild atopic dermatitis, which decreased the pH of the skin to 4.0 [38]. This study suggests that decreasing teat-skin pH with disinfection positively affects the maintenance of the acid mantle and the bacteriostatic properties of teat skin [37]. The additional advantages of pH 4.0–5.0 are related to the stability of curcumin, as it undergoes rapid autoxidation and alkaline hydrolysis at pH > 7.0, whereas its chemical structure remains significantly more stable under mildly acidic conditions at pH 3.0–5.0 [39].

In general, a 2% (*w*/*v*) chlorhexidine solution is the recommended skin antiseptic agent according to the evidence-based practice in infection control (EPIC) and CDC guidelines [40]. For teat dips, chlorhexidine at 0.35% [41] and 0.5% combined with glycerin [42], used as post-milking disinfectants for dairy cattle teats, showed good efficacy in reducing the rates of new natural and *S*. *aureus* intramammary infections, respectively. In this study, chlorhexidine was adjusted to 0.1% combined with glycerin in all the formulas, and all showed similar or better antimicrobial efficiencies than did TeatDip2, which is an iodine-based teat-dipping disinfectant from a local company in Thailand (Figure 2). TeatDip2, despite its relatively high labeled concentration of available iodine (3%), produced smaller inhibition zones against some of the tested bacteria than the experimental formulations did. These findings may reflect differences in formulation and iodine availability rather than iodine concentration alone. The antimicrobial activity of iodine-containing products depends on the availability of active iodine, while other formulation components may influence iodine release, stability, and diffusion through agar. In addition, the results of the agar well diffusion assay are influenced by the physicochemical properties and diffusion characteristics of the tested formulation [43]. Therefore, the inhibition zone results should be interpreted as the antibacterial activity of the formulations under specific in vitro assay conditions and should not be used to directly compare the overall antimicrobial effectiveness on the basis solely of the labeled iodine concentration. Quantitative antimicrobial assays, such as minimum inhibitory concentration (MIC) and time-kill assays, together with animal studies, are needed for a more comprehensive assessment of practical teat disinfection efficacy. The acceptable antimicrobial efficacy of the low chlorhexidine concentration formulas in this study might be attributable to the nanoformulations. Smaller, more homogeneously dispersed droplets from our nanoformulations provide greater surface area for interaction with the bacterial cell wall, improving the delivery and antibacterial activity of curcumin [44]. Compared with an ordinary curcumin formulation, curcumin nanoformulations have been reported to have better antimicrobial efficacy against various microorganisms, including *Staphylococcus aureus*, *Bacillus subtilis*, *Escherichia coli*, *Pseudomonas aeruginosa*, *Penicillium notatum*, and *Aspergillus niger* [45]. The tested chlorhexidine–curcumin formulations exhibited antibacterial activity against the mastitis pathogens evaluated. However, no significant differences were observed among CCN0, CCN0.1, and CCN0.5, indicating that no additional antibacterial effect was detected from the addition of 0.1% or 0.5% curcumin under the conditions tested. CCN0.9 showed greater inhibition against some streptococcal isolates; however, this finding cannot be attributed solely to its higher curcumin concentration because the composition and preparation of CCN0.9 differed from those of the other formulations. Further studies with appropriately matched controls and defined curcumin concentrations are needed to determine the specific contribution of curcumin and any potential interaction with chlorhexidine.

In this study, antibacterial efficacy against six mastitis pathogens was assessed. However, the diversity of organisms associated with bovine mastitis in practice is more extensive than this selection, and testing additional pathogens (such as other coagulase-negative staphylococci, *Klebsiella* spp., and mycoplasma) is advised to increase the applicability of these results. The limitation of this study is that the long-term stability of the nanoformulations was not evaluated. In addition, the antimicrobial activity was evaluated only by agar well diffusion assays under controlled in vitro conditions. This assay does not fully reproduce the physicochemical and biological conditions of the bovine teat or farm environment. In addition, the local tolerability, tissue compatibility, and potential toxicity of the formulations following teat application were not evaluated. From a practical perspective, the formulation approach may be suitable for further development because nanoemulsions can be manufactured using scalable emulsification technologies, including high-pressure homogenization [46]. Nevertheless, laboratory efficacy alone in this study does not establish farm-level feasibility. Therefore, further studies under defined storage conditions are needed to determine their physicochemical stability and retention of antibacterial activity over time. In addition, further in vivo studies are needed to assess both the antimicrobial performance and safety of these formulations under practical conditions.

## 5. Conclusions

In conclusion, the chlorhexidine–curcumin nanoformulations represent the antibacterial activity against the tested mastitis-associated bacteria under the conditions of this preliminary in vitro study. The exhibited activity was that it is equal to or exceeds the efficiency of commercial teat dipping against most pathogens tested. Based on the physicochemical characterization, the 0.5% chlorhexidine-curcumin (0.5%) nanoemulsion exhibited the optimal nanoscale characteristics. However, the present study was limited to in vitro screening using an agar well diffusion assay and did not evaluate teat-skin irritation, a formulation matrix, wound healing, or in vivo prevention of intramammary infection. Further studies are therefore required before the practical efficacy of these formulations can be established.

## Figures and Tables

**Table 1:** Composition ratios of the chlorhexidine-curcumin nanoformulations (CCNs).

Composition Ratio (%)	CCN0	CCN0.1	CCN0.5	CCN0.9
Curcumin	0	0.1	0.5	0.9
β-cyclodextrin	0.38	0.38	0.38	0.47
Liquid paraffin	10	10	10	0
DMSO	1	1	1	0
Acetone	0	0	0	4.7
Chlorhexidine	0.1	0.1	0.1	0.1
Glycerin	5	5	5	5

**Table 2:** Physicochemical characteristics of the chlorhexidine–curcumin nanoformulations (CCNs).

Characteristics	CCN0	CCN0.1	CCN0.5	CCN0.9
Size (nm)	2495.7 ± 52.5	101.3 ± 7.4	94.77 ± 0.3	215.6 ± 0.7
PDI (a.u.)	1.64 ± 0.04	1.07 ± 0.02	0.17 ± 0.03	2.1 ± 0.05
Zeta Potential (mV)	−2.2 ± 1.0	−38.4 ± 2.0	−46.57 ± 1.86	24.4 ± 0.67
Viscosity (cP)	0.896 ± 0.000	0.895 ± 0.002	0.896 ± 0.001	0.895 ± 0.002
pH	4.19 ± 0.282	4.24 ± 0.252	4.23 ± 0.329	4.68 ± 0.146
Conductivity (mS/cm)	0.049 ± 0.000	0.050 ± 0.000	0.083 ± 0.004	0.107 ± 0.001
%EE	0	88.6	97.7	93.9

The results are presented as the mean ± SD (n = 3). CCN0, CCN0.1, CCN0.5 and CCN0.9. PDI = polydispersity index, EE = entrapment efficiency.

## Data Availability

The data presented in this study are available from the corresponding author upon reasonable request.

## References

[1] Ruegg P.L. (2017). A 100-year review: Mastitis detection, management, and prevention. J. Dairy Sci..

[2] Rifatbegović M., Nicholas R.A.J., Mutevelić T., Hadžiomerović M., Maksimović Z. (2024). Pathogens associated with bovine mastitis: The experience of Bosnia and Herzegovina. Vet. Sci..

[3] Srithanasuwan A., Intanon M., Chaisri W., Suriyasathaporn W. (2023). In vitrobacterial competition of *Staphylococcus aureus*, *Streptococcus agalactiae*, and *Escherichia coli* against coagulase-negative staphylococci from bovine mastitis milk. Antibiotics.

[4] National Mastitis Council (2016). Current Concepts of Bovine Mastitis.

[5] Enger B.D., White R.R., Nickerson S.C., Fox L.K. (2016). Identification of factors influencing teat dip efficacy trial results by meta-analysis. J. Dairy Sci..

[6] Chaisri W., Pangprasit N., Srithanasuwan A., Intanon M., Suriyasathaporn W. (2019). Screening antimicrobial properties against mastitis pathogens of turmeric extract after combination with various antiseptics. Thai J. Vet. Med..

[7] Damasceno M.D., Gonçalves M.S., da Silva B.H.P., Carneiro G.B., Reis A.G., Pereira B.R., Costa A.C.T.R.B., Bueno Filho J.S.S., Dorneles E.M.S., Guimarães A.S. (2025). Susceptibility of mastitis-causing pathogens (*Escherichia coli* and *Staphylococcus aureus*) to disinfectants used as teat dipping. Vet. Microbiol..

[8] do Nascimento G.M., Rodrigues R.A., Brugnera H.C., Barbosa J.C., Favaron F.R., Rossi G.A.M., de Bragança C.R.S., Schocken-Iturrino R.P., de Ávila F.A., Cardozo M.V. (2025). Antimicrobial activity of teat antiseptic formulations based on plant extracts for controlling bovine mastitis: In vitroand in vivo evaluation. Vet. Sci..

[9] Tholang S., Singh R.S., Gupta D.K. (2023). A new herbal teat dip for preventing mastitis. Indian J. Anim. Health.

[10] Neijenhuis F., Barkema H., Hogeveen H., Noordhuizen J.P.T.M. (2001). Relationship between teat-end callosity and occurrence of clinical mastitis. J. Dairy Sci..

[11] Kumari A., Raina N., Wahi A., Goh K.W., Sharma P., Nagpal R., Jain A., Ming L.C., Gupta M. (2022). Wound-healing effects of curcumin and its nanoformulations: A comprehensive review. Pharmaceutics.

[12] Abd El-Hack M.E., El-Saadony M.T., Swelum A.A., Arif M., Abo Ghanima M.M., Shukry M., Noreldin A., Taha A.E., El-Tarabily K.A. (2021). Curcumin, the active substance of turmeric: Its effects on health and ways to improve its bioavailability. J. Sci. Food Agric..

[13] Hao M., Zhang C., Wang T., Hu H. (2025). Pharmacological effects, formulations, and clinical research progress of curcumin. Front. Pharmacol..

[14] Silva J.S.S.C., Barbaresco M.J., Rocha R.M., Eliseu M.G.F.R., de Andrade M., Dias L.D., Naves P.L.F. (2025). Chlorhexidine combined with curcumin-mediated photodynamic treatment effectively inhibits biofilm formation by clinical *Candida* isolates from the oral cavity. Photodiagn. Photodyn. Ther..

[15] Liu Y., Yin R., Tian Y., Xu S., Meng X. (2024). Curcumin nanopreparations: Recent advance in preparation and application. Biomed. Mater..

[16] Sohn S.I., Priya A., Balasubramaniam B., Muthuramalingam P., Sivasankar C., Selvaraj A., Valliammai A., Jothi R., Pandian S. (2021). Biomedical applications and bioavailability of curcumin—An updated overview. Pharmaceutics.

[17] Alshammari E.M. (2025). Curcumin-based biocompatible nanocarriers: A contemporary perspective in functional foods and biomedical applications. Discov. Nano.

[18] Schoeman C., van Niekerk S., Liebenberg W., Hamman J. (2024). Cyclodextrin inclusion complex and amorphous solid dispersions as formulation approaches for enhancement of curcumin’s solubility and nasal epithelial membrane permeation. Future J. Pharm. Sci..

[19] Mishra L., Gupta S. (2021). Fluconazole and curcumin loaded nanoemulsion against multiple drug resistance dermatophytes. Biomed. Pharmacol. J..

[20] Rachmawati H., Edityaningrum C.A., Mauludin R. (2013). Molecular inclusion complex of curcumin–β-cyclodextrin nanoparticle to enhance curcumin skin permeability from hydrophilic matrix gel. AAPS PharmSciTech.

[21] Balouiri M., Sadiki M., Ibnsouda S.K. (2016). Methods for in vitroevaluating antimicrobial activity: A review. J. Pharm. Anal..

[22] Bhawana, Basniwal R.K., Buttar H.S., Jain V.K., Jain N. (2011). Curcumin nanoparticles: Preparation, characterization, and antimicrobial study. J. Agric. Food Chem..

[23] Wahnou H., El Kebbaj R., Liagre B., Sol V., Limami Y., Duval R.E. (2025). Curcumin-based nanoparticles: Advancements and challenges in tumor therapy. Pharmaceutics.

[24] Qadir S., Imam S., Ali S.A., Ali F.R., Iffat W., Yousuf R.I., Usman R., Wahid S., Azhar I. (2026). Development of nanoemulsion formulation loaded with *Enteromorpha intestinalis* extract: Characterization and evaluation for topical use. PLoS ONE.

[25] Algahtani M.S., Ahmad M.Z., Ahmad J. (2022). Investigation of factors influencing formation of nanoemulsion by spontaneous emulsification: Impact on droplet size, polydispersity index, and stability. Bioengineering.

[26] Loftsson T., Brewster M.E. (1996). Pharmaceutical applications of cyclodextrins. Drug solubilization and stabilization. J. Pharm. Sci..

[27] Mashaqbeh H., Obaidat R., Al-Shar’i N. (2021). Evaluation and characterization of curcumin-β-cyclodextrin and cyclodextrin-based nanosponge inclusion complexation. Polymers.

[28] Ben Mihoub A., Acherar S., Frochot C., Malaplate C., Yen F.T., Arab-Tehrany E. (2021). Synthesis of new water-soluble β-cyclodextrin curcumin conjugates and in vitrosafety evaluation in primary cultures of rat cortical neurons. Int. J. Mol. Sci..

[29] Kang S., Kim M., Kim H., Hong J. (2024). Enhancement of solubility, stability, cellular uptake, and bioactivity of curcumin by polyvinyl alcohol. Int. J. Mol. Sci..

[30] Silva G.O., Oliveira P.A., Rodrigues M.C., Oliveira P.H., Silva S.W., Joanitti G.A., Carneiro M.L.B. (2026). Formulation and characterization of annatto oil and curcumin nanoemulsions with wound healing and antimicrobial potential. Proceedings.

[31] Singh P.A., Awasthi R., Pandey R.P., Kar S.K. (2025). Curcumin-loaded nanoemulsion for acute lung injury treatment via nebulization: Formulation, optimization and in vivo studies. ADMET DMPK.

[32] Mukherjee P., Baruah K.N., Srivastav P.P., Srivastava B., Karunanithi S. (2025). Characterization of nanoemulsion: Particle size, polydispersity index, zeta potential, thermal stability, and kinetic stability. Essential Oil Extraction from Food By-Products.

[33] McClements D.J. (2012). Nanoemulsions versus microemulsions: Terminology, differences, and similarities. Soft Matter.

[34] Sari T.P., Mann B., Kumar R., Singh R.R.B., Sharma R., Bhardwaj M., Athira S. (2015). Preparation and characterization of nanoemulsion encapsulating curcumin. Food Hydrocoll..

[35] Block S.S. (2001). Disinfection, Sterilization, and Preservation.

[36] Langan S.M., Irvine A.D., Weidinger S. (2020). Atopic dermatitis. Lancet.

[37] Fox L.K., Oura L.Y., Ames C.R. (2003). Short communication: Teat skin pH. J. Dairy Sci..

[38] Ng S.P., Bielfeldt S., Laing S., Danby S., Cork M.J.J. (2024). Effects of a pH-regulating emollient cream in mild atopic dermatitis patients with moderate localized lesions. Skin Pharmacol. Physiol..

[39] Nelson K.M., Dahlin J.L., Bisson J., Graham J., Pauli G.F., Walters M.A. (2017). The essential medicinal chemistry of curcumin. J. Med. Chem..

[40] Pratt R.J., Pellowe C.M., Wilson J.A., Loveday H.P., Harper P.J., Jones S.R., McDougall C., Wilcox M.H. (2007). epic2: National evidence-based guidelines for preventing healthcare-associated infections in NHS hospitals in England. J. Hosp. Infect..

[41] Oliver S.P., King S.H., Lewis M.J., Torre P.M., Matthews K.R., Dowlen H.H. (1990). Efficacy of chlorhexidine as a postmilking teat disinfectant for the prevention of bovine mastitis during lactation. J. Dairy Sci..

[42] Boddie R., Nickerson S., Adkinson R. (1997). Efficacies of teat germicides containing 0.5% chlorhexidine and 1% iodine during experimental challenge with *Staphylococcus aureus* and *Streptococcus agalactiae*. J. Dairy Sci..

[43] Morrill K.M., Scillieri Smith J.C., Dann H.M., Gauthier H.M., Ballard C.S. (2019). Evaluation of powdered 0.5% chlorhexidine acetate-based postmilking teat dip compared with a foamed 1% iodine-based postmilking teat dip under cold weather conditions in northern New York. J. Dairy Sci..

[44] Begum J.P., Sahu P., Vinode R., Patel A., Alomary M.N., Begum M.Y., Jamous Y.F., Siddiqua A., Al Fatease A., Ansari M.A. (2024). Antimicrobial nanoemulsion: A futuristic approach in antibacterial drug delivery system. J. Saudi Chem. Soc..

[45] Moghadamtousi S.Z., Kadir H.A., Hassandarvish P., Tajik H., Abubakar S., Zandi K. (2014). A review on antibacterial, antiviral, and antifungal activity of curcumin. Biomed. Res. Int..

[46] Kybartas M., Virgailis M., Ruzauskas M., Klimienė I., Šiugždinienė Ž., Merkevičienė L., Štreimikytė-Mockeliūnė Ž., Mockeliūnas R. (2023). A study on the stability and antimicrobial efficacy of a newly modeled teat dip solution containing chlorhexidine. Vet. Sci..

